# Supplementary material for: The Biological Activity of Fragmented Computer-Aided Design/Manufacturing Dental Materials before and after Exposure to Acidic Environment
Source: Medicina (Kaunas). 2023 Jan 3;59(1):104. doi: 10.3390/medicina59010104 (PMC9866959; doi:10.3390/medicina59010104)
Supplement: Supplementary file 1 [file medicina-59-00104-s001.zip › medicina-2053256-supplementary.pdf]

medicina

Supplementary Material

The Biological Activity of Fragmented Computer-Aided Design/Manufacturing Dental Materials before and after Exposure to Acidic Environment

Codruța Eliza Ille [1,2], Elena-Alina Moacă [3,4], Maria Suciu [5,*], Lucian Barbu-Tudoran [5,6], Meda-Lavinia Negruțiu [7,8] and Anca Jivănescu [1,2]

3.2. Impact of the CAD/CAM restorative materials powders on mitochondrial activity by means of MTT assay

Table S1. Mitochondrial activity percentage of BJ human cells

**Mitochondrial activity percentage of BJ human cells [%], after treatment with CAD/CAM restorative materials powders**			
	**Concentration tested [mg/mL]**		
**Sample denomination**	**1**	**0.5**	**0.2**
CS_A	59.24	96.18	84.71
CS_B	68.79	77.71	91.08
CS_C	38.22	50.32	44.59
SN_A	90.45	87.90	97.45
SN_B	89.81	86.62	99.36
SN_C	67.52	54.78	57.96
TC_A	61.15	66.88	77.07
TC_B	58.60	75.16	84.71
TC_C	35.67	40.13	68.79
Control		100	

Table S2. Mitochondrial activity percentage of HaCaT cells

**Mitochondrial activity percentage of HaCaT cells [%], after treatment with CAD/CAM restorative materials powders**			
	**Concentration tested [mg/mL]**		
**Sample denomination**	**1**	**0.5**	**0.2**
CS_A	86.71	78.73	78.15
CS_B	93.53	75.95	78.84
CS_C	84.51	84.28	75.38
SN_A	93.64	91.33	81.27
SN_B	80	80.46	49.13
SN_C	75.95	87.40	92.37
TC_A	95.95	69.13	77.80
TC_B	83.82	94.68	97.69
TC_C	98.27	100	98.27
Control		100	

3.3. Cytotoxicity evaluation of CAD/CAM restorative materials powders via LDH release method

Table S3. Cytotoxicity percentage of BJ cells

**Cytotoxicity percentage of BJ cells [mU/mL], after treatment with CAD/CAM restorative materials powders**			
**Concentration tested [mg/mL]**			
**Sample denomination**	**1**	**0.5**	**0.2**
CS_A	4.94	3.90	3.84
CS_B	4.19	4.05	3.78
CS_C	0.35	0.33	0.13
SN_A	3.93	3.68	3.93
SN_B	3.64	3.10	3.24
SN_C	0.31	0.30	0.02
TC_A	4.19	4.12	4.01
TC_B	3.61	3.42	3.32
TC_C	7.90	4.46	6.04
Control		4.04	

Table S4. Cytotoxicity percentage of HaCaT cells

**Cytotoxicity percentage of HaCaT cells [mU/mL], after treatment with CAD/CAM restorative materials powders**			
**Concentration tested [mg/mL]**			
**Sample denomination**	**1**	**0.5**	**0.2**
CS_A	13.61	12.93	17.87
CS_B	11.23	12.44	15.32
CS_C	5.11	5.30	7.37
SN_A	11.96	10.69	14.89
SN_B	7.31	5.59	6.91
SN_C	5.90	5.30	6.10
TC_A	15.03	13.88	16.02
TC_B	9.80	5.31	6.42
TC_C	0.55	0.43	0.23
Control		12.42	

3.4. NO production via Griess assay

Table S5. NO production of human BJ cells

NO production of human BJ cells [μg/mL], after treatment with CAD/CAM restorative materials powders			
	Concentration tested [mg/mL]		
**Sample denomination**	**1**	**0.5**	**0.2**
CS_A	0.020	0.016	0.014
CS_B	0.019	0.014	0.022
CS_C	0.017	0.014	0.014
SN_A	0.019	0.015	0.015
SN_B	0.017	0.016	0.014
SN_C	0.015	0.015	0.017
TC_A	0.020	0.013	0.013
TC_B	0.015	0.014	0.016
TC_C	0.016	0.016	0.018
Control		0.032	

Table S6. NO production of HaCaT cells

NO production of HaCaT cells [μg/mL], after treatment with CAD/CAM restorative materials powders			
	Concentration tested [mg/mL]		
**Sample denomination**	**1**	**0.5**	**0.2**
CS_A	0.027	0.022	0.021
CS_B	0.017	0.017	0.023
CS_C	0.018	0.016	0.018
SN_A	0.022	0.017	0.022
SN_B	0.019	0.018	0.015
SN_C	0.018	0.016	0.022
TC_A	0.021	0.020	0.019
TC_B	0.020	0.016	0.017
TC_C	0.018	0.016	0.014
Control		0.017	
